# Circulating Tumor DNA Testing for Homology Recombination Repair Genes in Prostate Cancer: From the Lab to the Clinic

**DOI:** 10.3390/ijms22115522

**Published:** 2021-05-24

**Authors:** Alessia Cimadamore, Liang Cheng, Francesco Massari, Matteo Santoni, Laura Pepi, Carmine Franzese, Marina Scarpelli, Antonio Lopez-Beltran, Andrea Benedetto Galosi, Rodolfo Montironi

**Affiliations:** 1 Section of Pathological Anatomy, School of Medicine, Polytechnic University of the Marche Region, United Hospitals, 60126 Ancona, Italy; a.cimadamore@staff.univpm.it (A.C.); laura.pepi27@gmail.com (L.P.); m.scarpelli@staff.univpm.it (M.S.); 2 Department of Pathology and Laboratory Medicine, School of Medicine, Indiana University, Indianapolis, IN 46202, USA; liang_cheng@yahoo.com; 3 Medical Oncology, IRCCS Azienda Ospedaliero-Universitaria di Bologna, Via Albertoni 15, 40138 Bologna, Italy; fmassari79@gmail.com; 4 Oncology Unit, Macerata Hospital, 62100 Macerata, Italy; mattymo@alice.it; 5 Department of Specialist Clinical Science and Odontostomatology, Urology Division, Polytechnic University of the Marche Region, United Hospitals, 60126 Ancona, Italy; carminefranzese_@msn.com (C.F.); galosiab@yahoo.it (A.B.G.); 6 Department of Morphological Sciences, Cordoba University Medical School, 14071 Cordoba, Spain; em1lobea@gmail.com

**Keywords:** circulating tumor DNA, prostate cancer, metastatic castration-resistant prostate cancer, homology recombination repair genes, BRCA1, BRCA2, ATM, PARP inhibitors

## Abstract

Approximately 23% of metastatic castration-resistant prostate cancers (mCRPC) harbor deleterious aberrations in DNA repair genes. Poly (ADP-ribose) polymerase (PARP) inhibitors (PARPi) therapy has shown improvements in overall survival in patients with mCRPC who harbor somatic and/or germline alterations of homology recombination repair (HRR) genes. Peripheral blood samples are typically used for the germline mutation analysis test using the DNA extracted from peripheral blood leucocytes. Somatic alterations can be assessed by extracting DNA from a tumor tissue sample or using circulating tumor DNA (ctDNA) extracted from a plasma sample. Each of these genetic tests has its own benefits and limitations. The main advantages compared to the tissue test are that liquid biopsy is a non-invasive and easily repeatable test with the value of better representing tumor heterogeneity than primary biopsy and of capturing changes and/or resistance mutations in the genetic tumor profile during disease progression. Furthermore, ctDNA can inform about mutation status and guide treatment options in patients with mCRPC. Clinical validation and test implementation into routine clinical practice are currently very limited. In this review, we discuss the state of the art of the ctDNA test in prostate cancer compared to blood and tissue testing. We also illustrate the ctDNA testing workflow, the available techniques for ctDNA extraction, sequencing, and analysis, describing advantages and limits of each techniques.

## 1. Introduction

In 2015, The Cancer Genome Atlas Research Network reported findings from 333 primary prostate cancers which resulted in the identification of 19% of primary tumors with mutations in DNA repair genes, including 3% in the homologous recombination repair (HRR) gene, BRCA2 [1]. Exome sequencing of 150 metastatic biopsies found that 23% of metastatic prostate cancers carry alterations in the genes critical for DNA repair, again, involving homologous recombination repair genes (BRCA2, ATM, and BRCA1), as well as mismatch repair genes (MLH1 and MSH2) [2]. Alterations in these genes include base pair substitutions, deletions, insertions, copy number variations (CNV), and selected rearrangements [3]. Men with germline mutations are more likely to harbor intraductal/ductal histology (48% vs. 12%, *p* < 0.01) and lymphovascular invasion (52% vs. 14%, *p* < 0.01), while neither prostate cancer family history nor other cancers in a given family were statistically associated with a positive germline test [4,5]. Genomic testing in advanced PCa patients is currently recommended in a specific setting (Table 1) [6,7,8,9].

The rationale behind genetic testing of PCa patients is that metastatic castration-resistant PCa (mCRPC) with DNA repair genes alteration may be a potential candidate for target therapy with PARP inhibitors. Recent trials with PARP inhibitors in patients with mCRPC have demonstrated significant improvements in overall survival (OS) in patients harboring germline and/or somatic alterations in DNA repair genes compared to enzalutamide and abiraterone. 

In brief, the PROfound study is a Phase 3 multicenter trial aimed at evaluating the PARP inhibitor olaparib in men with mCRPC who had disease progression while receiving a new hormonal agent (e.g., enzalutamide or abiraterone). Men who had an alteration in genes involved in the homologous recombination repair pathway were divided in two cohorts: cohort A (245 patients) with at least one alteration in BRCA1, BRCA2, or ATM, and cohort B (142 patients) with an alteration in any of the 12 other HHR genes. In cohort A, significant improvement in median rPFS (7.4 vs. 3.6 months) and in OS (19 vs. 14 months) were reported. In cohort B, the median duration of OS was 14.1 months with olaparib and 11.5 months with control therapy [10,11]. Exploratory gene-by-gene analysis presented at ASCO 2021 showed that for BRCA-mutated patients, ORR was 43.9% vs. 0% and median OS was 20.1 vs. 14.4 months for olaparib and enzalutamide or abiraterone, respectively. Concerning the other genes analyzed, olaparib monotherapy also resulted in a higher ORR in patients with alterations in CDK12 (5.9% vs. 0%) but not ATM (10.0% vs. 10.0%), and no significant difference in rPFS or OS was reported among patients with ATM or CDK12 alterations [12].

The TRITON2 was a Phase II study of rucaparib in mCRPC patients with HRR gene alterations [13]. Fifty-two percent of the 98 patients had a confirmed PSA response (≥50% decrease from the baseline) and 44% of the 57 patients with measurable disease had a confirmed RECIST partial (22 patients) or complete response (three patients). A greater PSA response rate was observed in patients with a BRCA2 alteration. Furthermore, in this trial, limited radiographic/PSA responses to PARP inhibition were reported in men with alterations in ATM, CDK12, or CHEK2, while responses have been confirmed in other DDR genes such as PALB2, FANCA, BRIP1, and RAD51B [14].

These results led to an FDA approval of two PARP inhibitors in 2020, olaparib and rucaparib, for mCRPC with DNA repair gene alterations. These drugs represent the first approval in the history of PCa therapy that requires a specific molecular biomarker for select patients. Molecular testing can be performed on whole blood, on formalin-fixed paraffin-embedded (FFPE) tumor tissue, or circulating tumor DNA extracted from plasma samples [15]. 

## 2. Tissue Testing vs. Whole Blood Testing

The main difference between tissue and blood-based testing is the type of mutation detected. Blood testing is used to detect significant genomic rearrangements to assess familiar risk and it is feasible in one hundred percent of the cases; however, it does not identify mutations of somatic origin. Tissue testing can detect both somatic and germline mutations [16]. Patients with a detected tissue mutation are referred to germline testing to determine if the mutation is germline or somatic. Germline testing requires genetic counselling, similarly to blood testing [15]. 

The advantages of blood testing are the ease of obtaining samples, minimal invasiveness, and repeatability. Tissue testing can be performed on prostate samples (both biopsies and surgical specimens) and on metastatic deposits. In the latter case, obtaining tissue from a metastatic site is an invasive procedure characterized by high cost and morbidity for patients. Moreover, in the specific case of PCa patients, bone is a frequent site of metastasis. DNA extracted from a bone biopsy, if undergone through a decalcification process, can be qualitatively very poor and molecular test is likely to fail [16]. 

DNA quality is another important parameter to consider when choosing the best-performing test. It depends on purity of the sample and fragmentation of DNA. DNA quality is high in blood and variable in FFPE tissue [17]. Factors that can lower DNA quality are in common with other molecular tissue tests, i.e., presence of necrosis, high infiltration of inflammatory cells, aging-related degradation, poor fixation in formalin, cauterized tissue [18]. Nonetheless, the reliability of tumor testing in diagnosing or excluding germline mutations is questionable. A recent study reported that in patients with no germline pathogenic BRCA1/2 mutation, tumor tests were 100% concordant with no false positive results. However, in patients who harbored a germline pathogenic BRCA1/2 mutation, only 70% of these germline mutations were identified with tumor testing, while 30% of germline mutations were missed, due to either technical or interpretative errors [19]. 

## 3. Biology of ctDNA

Circulating cell-free DNA (cfDNA) in blood acquired from plasma using a simple blood test provides repeated serial access to tumor DNA as a minimally invasive “liquid biopsy.” Liquid biopsy includes cell-free nucleic acid, circulating tumor cells, extracellular vesicles, metabolites, and proteins [20,21,22]. 

The total amount of circulating DNA found in blood plasma is called circulating free DNA and it accounts for the total DNA released by normal and tumor cells. It can be present in healthy subjects and its concentration can be increased in case of stroke, trauma, myocardial infarction, and autoimmune disease [23,24,25]. Concentration of cfDNA is much higher in advanced cancer patients compared to healthy individuals [26,27].

Circulating tumor DNA refers only to the plasma DNA that originates specifically from tumors, primary site, metastases, and even circulating tumor cells. It can represent from 0.01% up to 90% of the total free DNA. Its concentration differs between patients depending on location, size, vascularity of the tumor, cancer stage, cellular turnover, and response to therapy. The level of ctDNA correlates with disease progression and is higher in metastatic tumor than in localized diseases [28,29,30,31].

Circulating cell-free DNA can be released by cells through passive release or active secretion. The first way occurs through cell death that can happen by apoptotic process or necrosis. The two ways differ in the length of DNA fragments. During apoptosis, the enzymatic cleavage of DNA produces DNA fragments that are still wrapped around single nucleosomes and the length plus linker is around 166 base pairs. In case of necrosis, larger fragments are shed into the circulation up to one thousand base pairs [32]. A small fraction of ctDNA originates from CTC by passive release. A minority of ctDNA is released by active secretion of extracellular vesicles, such as exosomes and prostasomes containing pieces of DNA around 150–250 bp [33]. 

## 4. Circulating Tumor DNA in Prostate Cancer

Tissue testing is currently the principal test used for the analysis of tumor genomic profiles. However, concerning PCa patients, in the main trials in which tissue testing was performed before enrollment, about 30% of tests failed due to pathology review failure, DNA extraction failure, or failure after DNA extraction [11,34,35,36]. Consequently, the possibility of assessing molecular alterations using ctDNA is gaining attention in the clinical and laboratory community [37]. 

Shedding of ctDNA differs between untreated patients, androgen deprivation (ADT)-treated patients, and mCRPC patients. Plasma-extracted cfDNA sequencing was successful in 52/53 patients with de novo metastatic castration-sensitive PCa. Before ADT initiation, 74% of the patients had detectable ctDNA, while in the patients who received ADT prior to blood collection, only 59% had detectable ctDNA. The ctDNA fraction was significantly lower than among treatment-naïve patients (mean, 6.7% vs. 23%; median, 1.0% vs. 11%; *p* = 0.02). The reduction in the ctDNA fraction was more pronounced after one week of ADT [38,39]. No relationship between the ctDNA fraction and PSA, Gleason grade, or age was reported. 

A CtDNA fraction above 30% was strongly associated with poor response to therapy with enzalutamide or abiraterone even after adjustment for other clinical prognostic factors. Cell-free DNA concentration changes after PARP inhibitor therapy are predictive biomarkers of response; indeed, a ≥50% fall in cfDNA concentration after eight weeks of therapy was independently associated with longer OS. 

Compared to ctDNA, tissue testing may miss tumor evolution through therapy and tumor heterogeneity, especially if the tumor test is performed on the primary tumor site. Circulating tumor DNA can be used to detect resistance mutations and is better representative of tumor heterogeneity.

Concordance between tissue testing and ctDNA has proven to be very high for what concerns the detection of deleterious alterations in BRCA1 or BRCA2 with a positive percent agreement of 88% and negative percent agreement of 95%. Some discordance is expected based on biological differences and sampling times between tumor tissues and plasma samples [11]. The concordance results stratified by variant show that positive agreement is high for substitutions and indels, 92% and 95%, respectively, while it is much lower for rearrangements and copy number loss. The negative agreement is almost 100% [40].

A retrospective study conducted in mCRPC patients profiled with a 70-gene cfDNA NGS panel (Guardant 360™) reported ≥ one alteration in 94% of 514 patients. A higher number of ctDNA alterations was associated with shorter time to treatment failure (HR = 1.05, *p* = 0.026) in patients treated with chemotherapy or androgen inhibitors. Serial ctDNA profiling of 64 patients revealed the evolution of alterations in AR, BRCA1, and BRCA2 following therapy [41]. 

The most recent and largest study on ctDNA in advanced PCa demonstrated high concordance between alterations identified by liquid and tissue biopsy and, moreover, proved the added value of liquid biopsy [42]. Out of the 3334 plasma samples from patients with mCRPC (including 1674 screening samples from TRITON2/3), 94% had detectable ctDNA (median ctDNA fraction, 7.5%; interquartile range, 0.8–34%). In 79.5% of all the patients, liquid biopsy identified at least one genomic alteration predicted to have deleterious/oncogenic effects on protein function: Tp53 (46%), AR (42%), BRCA2 (7.5%), BRCA1 (1.4%), PI3K/AKT/mTOR (14%), WNT/β-catenin pathway genes (17%), RAS/RAF/MEK (5%), MSI-H status (1.4%). Regarding BRCA mutations, concordance between tissue and ctDNA, 67 (8.0%) BRCA1/2 alterations were detected concordantly in tissue and liquid biopsy, 5 (0.6%)—exclusively in tissue biopsy, and 20 (2.4%)—exclusively in liquid biopsy. Of the five patients with BRCA alterations detected only in tissue biopsy, four had a ctDNA fraction below 1%. The 20 cases detected only in ctDNA analysis may represent alterations acquired after tissue specimen collection. In presence of a germline BRCA alteration, ctDNA analysis was able to detect it in 100% of the cases. It is worth noticing that in the near future, other genetic alterations such as mutations in AR or in PIK3CA/AKT1/PTEN should be included in molecular analysis since target therapy is currently under investigation (Table 2) [34,35,43,44,45,46]. 

## 5. Pros and Cons of ctDNA Testing

Both somatic and germline mutations can be detected by ctDNA testing. As for blood testing, advantages of ctDNA are the readiness in obtaining samples and repeatability during therapy or progression of disease. Turnaround time is largely in favor of ctDNA testing, 1–2 weeks, compared to around one month for tissue and 2–4 weeks for blood testing [47,48,49]. 

The main drawbacks of ctDNA are DNA quantity and quality [50]. DNA quantity is usually very low, depending on the fraction of tumor DNA in cell-free DNA. Lower concentrations are reported, particularly at early stages, in localized tumors, where the amount of DNA shedding is a minimal part of the whole cfDNA [51].

A negative result does not rule out the presence of a mutation in the patient’s tumor. In this case, patients should be referred to an FDA-approved tumor tissue test, if feasible. In case of the gene alterations reported, the test does not distinguish between germline and somatic ones. If a reported alteration is suspected to be germline, confirmatory testing should be considered in the appropriate clinical context.

For specific ctDNA tests, false positive rate has been evaluated in healthy controls. The detection rate for unique short variants resulted to be around 0.82%. Across 30,622 short variants, 58 variants had a detection rate of greater than 5%. A false positive result may be derived by non-tumor somatic alterations, such as clonal hematopoiesis of indeterminate potential (CHIP) [52,53]. Genes with alterations that potentially derive from CHIP include, but are not limited to, the following: ASXL1, ATM, CBL, CHEK2, DNMT3A, JAK2, KMT2D (MLL2), MPL, MYD88, SF3B1, TET2, TP53, and U2AF1 [54,55,56].

The level of ctDNA is critical for the performance of the test. The ctDNA fraction can vary during therapy and closely correlate with the tumor response. It is recommended that blood samples be drawn shortly before chemotherapy or at least two weeks after the previous treatment (Table 3) [57]. 

## 6. Workflow of ctDNA Testing

The practical workflow can be described in three steps: plasma sample preparation, ctDNA extraction, ctDNA analysis. 

### 6.1. Sample Collection, Plasma Separation, and Storage

Preparation of cfDNA samples involves sample collection, plasma separation, and storage. Sample collection consists in a venipuncture and requires a minimum of 2 mL of plasma. Blood can be collected in EDTA tubes or cfDNA-stabilizing tubes that are tubes with special preservatives that can be used for long-term storage and when plasma separation is not performed in a short time after blood collection [58,59]. 

When using EDTA tubes, separation of plasma from blood should be performed in a short time since the half-time of ctDNA is only about two hours in standard EDTA tubes, whereas with stabilizing tubes, plasma separation can be delayed. Plasma separation is obtained by simply centrifuging whole blood twice: once to separate plasma and the second time to remove cells and cell debris [60,61]. 

With a Streck tube, steady cfDNA quality can be obtained after up to one week of storage, independently of the temperatures [62]. However, even with these special tubes, an increased level of DNA has been reported after seven days of storage at 4 °C and room temperature [63,64]. 

The increase in DNA levels is due to the release of DNA from blood cell lysis, in particular, from white blood cells, and this can impair the performance of the test since tumor DNA present in plasma is diluted in non-tumor DNA [65,66]. 

### 6.2. DNA Extraction

There are various methods that can be used to extract cfDNA [67]:

- The magnetic enrichment method is one of the most used; it is based on positively charged magnetic beads that bind the negatively charged phosphate backbone of DNA. It is ideal for automation of high-throughput processing as they eliminate the need for centrifugation and other time-consuming steps, and it is more efficient in the recovery of short ctDNA fragments as compared to the silica-based membrane and conventional methods [68].
- The phenol chloroform method is a conventional method of extraction and ensures a higher yield compared to other DNA extraction kits. However, DNA purity is lower, resulting in a lower efficiency of downstream analysis [69].
- Silica column-based enrichment uses the affinity of DNA molecules to the silica membrane to bind DNA; it is fast, easy to perform, and economical. This method is the principle of Qiagen extraction kits and it is characterized by high recovery but partial loss of small fragments [70].
- In the polymer mediated-enrichment, DNA is captured by a polymer that is then centrifuged and dissolved in a special buffer. This technique reduces the sample volume; however, lower DNA yields are obtained compared to other methods.

Automated cfDNA extraction has been less evaluated than manual approaches. Besides high throughput and low hands-on time, the use of automated methods reduces variability and the risk of sample-to-sample contamination, though they generate somewhat lower cfDNA yields than manual extraction [71,72]. Recently, results from a comparison study on four commercially available (semi-)automated cfDNA extraction protocols have been published [73]. The same plasma samples were processed with Qiagen, Promega, Thermo, and Stratec extraction methods in order to evaluate their suitability for further NGS analysis. Concentrations and total amounts of cfDNA were highest for Qiagen (filter-based) and Promega (bead-based) protocols, showing the best read length profiles after sequencing.

### 6.3. DNA Sequencing and Analysis

After DNA extraction from plasma, the next step is DNA sequencing. Isolated DNA fragments are thus converted to the library by ligation to sequencing adapters containing specific sequences designed to interact with the NGS platform, either the surface of the flow cell (Illumina) or beads (Ion Torrent). The next step involves clonal amplification of the library by either cluster generation (Illumina) or microemulsion PCR (Ion Torrent). The final step generates the actual sequence via the chemistries for each technology. One difference between the two technologies is that Illumina allows sequencing from both ends of the library insert (i.e., paired-end sequencing). 

#### 6.3.1. Targeted vs. Untargeted Approach

According to the purpose of DNA analysis, two types of approach are possible: a targeted approach and a genome-wide approach. The targeted approach requires detailed information about the tumor genome and lower concentrations of input cfDNA. The targeted approach is extremely sensitive, as mutations can be detected at an allele frequency as little as 0.01%, highly specific, easier to interpret and implement in routine clinical practice, fast, and cost-effective.

On the other hand, in the untargeted or genome-wide approach, all the codifying parts of the genome (whole exome sequencing) or even the whole genome is sequenced and analyzed. This strategy has the ability to identify novel changes during tumor treatment and does not require prior information about the primary tumor genome. Nevertheless, high concentrations of ctDNA are required for reliable reconstruction of tumor-specific genome-wide changes, the sensitivity ranges from 5 to 10%. Nowadays, it is still an expensive technique with long turnaround times that requires skilled bioinformaticians for data analysis and interpretation [74]. 

In the case of PCa, for therapeutic purposes, a targeted approach is recommended since a panel of genes is sufficient to cover the current indications of PARP inhibitors and potentially biomarker-driven therapy (AKT, PTEN, PI3KCA) [28,75,76]. The preferred method to perform the PCa gene panel is a next-generation sequencing (NGS)-based platform [77]. While in some tumors PCR-based approach is still an option, when it comes to BRCA genes, NGS methods are more appropriate. Different types of mutations and alterations have been found so far, ranging from deletion, insertion, missense, and stop codon mutation to significant rearrangements [78,79]. New variants are continuously identified and a variable number, up to 20%, of variants of unknown significance (VUS) has been reported in the literature. 

**Figure 1:**
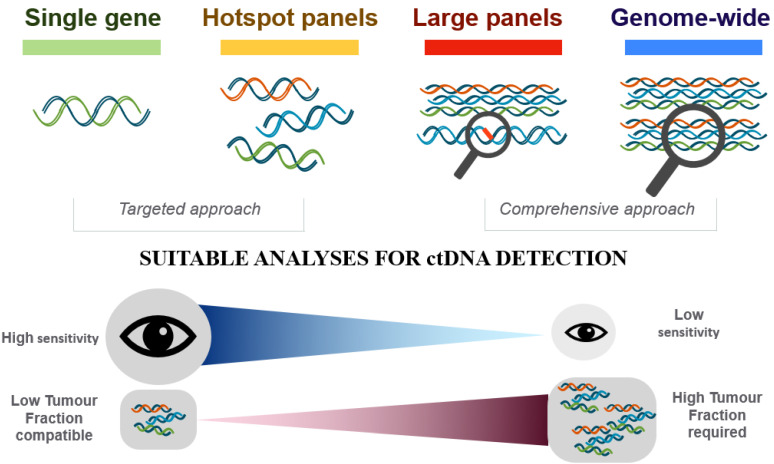
Targeted vs. genome-wide approach in ctDNA analysis.

Targeted NGS panels can range from hotspot panels focused on individual codons to more comprehensive panels that include the coding regions of hundreds of genes. Targeted panels can be specific for therapy selection while extensive panels can be adopted for research purpose (Figure 1).

In the targeted approach, it is essential to enrich our sample of our target of interest, and this can be done with an enrichment method capable of capturing selective genomic regions of interest for NGS. The two enrichment methods are hybridization-based or amplicon-based. 

In the hybridization method, DNA is hybridized to single-stranded oligonucleotides that are called probes or baits designed to target a specific region of interest. The probes capture the DNA of interest and can be recovered using streptavidin-coated magnetic beads since they are biotinylated. Amplicon-based enrichment uses primers designed to target a specific region of interest that is amplified by PCR, generating thousands of amplicons. To each amplicon, an adaptor with a unique identifier is attached to further amplify DNA in parallel single-plex reactions.

#### 6.3.2. Targeted NGS Techniques

Targeted NGS techniques used for ctDNA analysis currently adopted are cancer personalized profiling by deep sequencing (CAPP-Seq) and tagged amplicon deep sequencing (TAM-Seq). In the first technique, ctDNA is detected using “selectors” (specific probes) that selectively capture a set of exonic and intronic regions known to be recurrently affected in a particular cancer type [80]. 

Hybridization of “selectors” consisting of biotinylated DNA oligonucleotides that are complementary to previously defined recurrent mutated areas of interest is followed by deep sequencing. Deep sequencing is considered the first approach to detect mutations at an allele frequency as low as <0.2% by sequencing the target regions with high coverage (>10,000×) [81,82,83]. The main disadvantages of deep sequencing are the extremely high read depth that is required in order to detect mutations at low allele frequency and the resulting high sequencing costs.

In TAm-Seq (tagged amplicon deep sequencing), special primers to amplify a specific region of interest are designed. It is based on a two-step amplification process: the primers are first used to bind to the template during the preamplification step to amplify the original signal; then, the templates undergo individual amplification for purification. In this phase, a microfluidics system is used to attach adaptors with a unique identifier to each amplicon to further amplify DNA in parallel single-plex reactions. TAm-seq can identify mutations ~2% MAF with a sensitivity over 97%; the enhanced version of TAm-Seq named eTAm-Seq™ can detect MAF as low as 0.25% with a sensitivity of 94% [84,85].

To improve sensitivity of NGS, further techniques have been developed. In this context, it is worth mentioning Safe-Seq and Duplex-Seq. 

Safe-Seq assigns a unique identifier (UID) to each template molecule before amplification. Amplification of each uniquely tagged template molecule creates UID families so that many daughter molecules with an identical sequence are generated. If a mutation preexisted in the template molecule used for amplification, that mutation should be present in every daughter molecule containing that UID. On the other hand, if a mutation does not appear in most of the same UID-connected sequences, it is likely to be induced by other errors. In this way, Safe-Seq reduces sequencing errors at least 70-fold and has a sensitivity as high as ~98% for detecting tumor mutations [86,87].

Contrary to conventional sequencing technologies that sequence only a single strand of DNA, duplex sequencing examines both strands of DNA and scores mutations only if they are present on both strands of the same DNA molecule as complementary substitutions. DS uses adapters ligated onto the template to uniquely tag each DNA fragment in a plasma cfDNA sample. PCR amplification creates fragment families characterized by unique combinations of barcodes at both the 5′ and 3′ ends. A true variant will appear in all the reads within a family, while polymorphisms within a family represent sequencing and amplification errors that can be identified and removed by generating consensus sequences. As the two strands are complementary, true mutations are found at the same position in both strands (duplex consensus sequences) whereas PCR or sequencing errors result in mutations in only one strand and can thus be discounted as technical errors [88,89]. 

Both Safe-Seq and Duplex-Seq are characterized by a very low error rate due to duplex tagging and tags with unique identifiers. However, this strategy needs a relatively higher depth of sequencing to ensure adequate representation of each family and specialized informatics pipelines.

## 7. NGS ctDNA Testing Validation in the Laboratory

It is necessary to validate the ctDNA test according to the guidelines released by the Blood Profiling Atlas in Cancer Consortium (BloodPAC). The Consortium released a series of general protocols for analytical validation of NGS-based ctDNA assays [90]. As for any other test, conventional parameters need to be addressed: reference range, limit of blank (LoB), limit of detection (LoD), limit of quantification (LoQ), analytical accuracy, reproducibility, interfering substances, and specimen stability. This procedure is aimed at developing a range of global standards to harmonize clinical laboratory practices, ensure robustness, and define the level of stringency or the workflow challenges presented in the development of highly sensitive ctDNA-based NGS tests. Indeed, unlike tissue-based tests, ctDNA testing is characterized by a low concentration of the analyte (down to several mutant molecules per 10-mL blood collection tube) and thus requires an increased use of contrived specimens for validation. Moreover, presence of non-tumor-derived mutations as an endogenous analyte, i.e., of germline or CHIP mutations, poses another challenge.

## 8. Conclusions and Future Directions

Genomic testing in advanced PCa patients is currently recommended in specific settings. Molecular diagnostics should be performed by a certified (accredited) institution using a standard NGS procedure. Whole blood, tissue, ctDNA testing each have their own benefits and limitations. Genetic testing using ctDNA is a valid option as proven by multiple clinical trials and it received approval by the FDA in 2020. Moreover, longitudinal ctDNA testing can be performed to detect new alterations, even the resistance mutations that can emerge during disease progression. Tissue testing alone may not be sufficient and can fail in 30% of cases, whereas ctDNA testing can be performed as an alternative to tissue tests or even as a complementary test. Combined use of ctDNA and primary tissue is ideal for assessing the molecular subtype and pave the way for targeted therapy implementation in a precision oncology context. Third-generation sequencing technologies such as PacBioScience and Oxford Nanopore allow capturing sequence information during the replication process of the target DNA. These technologies can also help to detect intermediate large chromosomal aberrations which are of deep impact for PCa prognosis and seem to contribute to a progressive and stem cell-like phenotype of PCa [91,92]. 

These new technologies require minimal sample processing, therefore, smaller instruments have been being designed over the years until the recently commercialized portable gene sequencer of the size of a USB flash drive that can be used readily by connecting to a laptop [93]. However, the main drawback is the high error rate of these sequencers. In the future, the improvements of these systems along with the decrease of their costs will allow generating more comprehensive and easy-to-read data.

## Figures and Tables

**Table 1:** Recommendations for germline and somatic genomic testing in advanced PCa.

Recommendations	Germline Testing	Tumor Testing
NCCN [6]	Recommend germline testing for any patients with PCa and Ashkenazi Jewish ancestry or strong family history of cancer and for all patients with high-risk localized or metastatic PCa	Recommend tumor testing for HRRm and consider tumor testing for MSI/MRR alterations for all M1 patients Consider testing at earlier stages
ESMO [7]	Germline testing for BRCA2 and other DDR associated with cancer predisposition syndromes is recommended in patients with a family history of cancer and should be considered in all patients with metastatic prostate cancer	Consider tumor testing for HRR genes and MMR defects (or MSI) in patients with mCRPC
EAU [8,9]	Consider germline testing for patients with: metastatic PCa; high-risk PCa and a family member diagnosed with PCa at the age <60 years; multiple family members diagnosed with castration-sensitive PCa at the age <60 years or a family member who died from PCa; family history of high-risk germline mutations or a family history of multiple cancers on the same side of the family	All metastatic patients should be offered somatic genomic testing for HRR and MMR defects, preferably on metastatic carcinoma tissue, but testing on primary tumor may also be performed. Alternatively, but still less common, genetic testing on circulating tumor DNA (ctDNA) is an option

**Table 2:** Summary of the recently published papers that used ctDNA sequencing for the detection of HRR alterations in PCa.

Study	Objective	No. of Samples	Method	Results
Wyatt, A, et al. J Natl Cancer Inst 2017 [40]	To compare ctDNA alterations with the matched tissue and quantify the concordance	45 samples at the time of metastatic tissue biopsy	Targeted sequencing across 72 clinically relevant genes	75.6% had a ctDNA proportion greater than 2% of the total cfDNA; all the somatic mutations identified in matched metastatic tissue biopsies were concurrently present in ctDNA; concordance of 88.9% for individual gene CNA
Vandekerkhove, G., et al. Eur Urol 2019 [38]	To determine ctDNA abundance at de novo mCSPC diagnosis	53 mCSPC	Targeted sequencing strategy capturing the exons of 73 PC driver genes	Median ctDNA fraction was 11% (range, 0–84%) among the untreated patients but was lower (1.0%; range, 0–51%) among the patients after ADT; concordance for mutation detection in the matched samples was 80%
Sonpavde, G., et al. Cancer 2019 [41]	To evaluate association between cfDNA alterations and outcomes and evolution with therapy in mCRPC patients	514 mCRPC	Guardant360 assay	94% had ≥1 ctDNA alteration, higher numbers of ctDNA alterations, AR alterations, and amplifications of MYC and BRAF were associated with worse failure-free survival and/or OS
Matsubara, N, et al. 2021 JCO [46]	PROfound trial, cohort A (BRCA/ATM alteration-positive by tissue testing)	181 mCRPC	FoundationOne Liquid CDx assay	139/181 (76.8%) patients had a ctDNA result reported; 111/139 (79.9%) patients reported a BRCA/ATM GA; and 28/139 patients did not have either due to lack of ctDNA shedding from the tumor or ctDNA levels below the sensitivity of the assay
Tukachinsky, H., et al. Clin Cancer Res 2021 [42]	To evaluate the landscape of GA detected in ctDNA and assess concordance with tissue-based CGP	3334 patients with mCRPC	FoundationOne Liquid CDx assay	94% had detectable ctDNA with a median ctDNA fraction of 7.5%; of the 72 patients with BRCA1/2 mutations detected in tissue biopsy, 67 (93%) were also identified by ctDNA (100% for germline variants); in 20 patients, BRCA1/2 GA were identified using ctDNA but not tissue testing

GA: genomic alterations; ctDNA: circulating tumor DNA; mCSPC: metastatic castration-sensitive prostate cancer; mCRPC: metastatic castration-resistant prostate cancer; CPG: comprehensive genomic profiling; CNA: copy number alterations; ADT: androgen deprivation therapy.

**Table 3:** Benefits and limitations of tissue, ctDNA, and blood-based HRR gene tests.

	Tissue	ctDNA	Blood
Types of mutations detected	Somatic and germline		Germline only
Sample quality	DNA quantity: medium DNA quality: low	DNA quantity: low DNA quality: variable	DNA quantity: high DNA quality: high
Turnaround times (TAT)	~2–8 weeks	~1–2 weeks	~2–4 weeks
Genetic counselling	Patients with a positive mutation are referred for germline testing to determine whether the mutation is somatic or germline		Requires counselling
Testing benefits	Gold standard for tumor analysis High clinical sensitivity Archival tissue for tumor histology (Gleason scoring and/or ISUP grading may already be available and provides an option for testing)	Easy to obtain samples Better representative of tumor heterogeneity and metastatic deposits than primary biopsy Minimally invasive and easily repeatable	Easy to obtain samples Assesses familial risk Analysis feasible in 100% of cases Minimally invasive and easily repeatable Blood testing is currently used to detect significant genomic rearrangements
Testing limitations	May miss within-tumor genetic heterogeneity Obtaining samples from metastases is an invasive procedure and may be challenging Around 31% of tests fail due to pathology review failure, DNA extraction failure, or failure after DNA extraction	Low concentrations of circulating ctDNA Highly sensitive tests are required May result in false positives Testing is limited by the availability of an adequate amount of ctDNA, particularly at early stages	Does not identify patients with mutations of somatic origin or capture the potentially changing genetic profile of disease progression Does not detect HRRm of somatic origin
